# Efficacy of a Multimodal Digital Behavior Change Intervention on Lifestyle Behavior, Cardiometabolic Biomarkers, and Medical Expenditure: Protocol for a Randomized Controlled Trial

**DOI:** 10.2196/50378

**Published:** 2024-10-30

**Authors:** Sakeina Howard-Wilson, Jack Ching, Sherri Gentile, Martin Ho, Alex Garcia, Didem Ayturk, Peter Lazar, Nova Hammerquist, David McManus, Bruce Barton, Steven Bird, John Moore, Apurv Soni

**Affiliations:** 1 Department of Medicine University of Massachusetts Chan Medical School Worcester, MA United States; 2 Google LLC Mountain View, CA United States; 3 Clinician Experience Office University of Massachusetts Memorial Health University of Massachusetts Chan Medical School Worcester, MA United States; 4 Department of Population and Quantitative Health Sciences University of Massachusetts Chan Medical School Worcester, MA United States

**Keywords:** health behavior, fitness, digital devices, lifestyle change, cardiovascular disease, chronic disease, physical activity, nutrition, sleep, mindfulness

## Abstract

**Background:**

The US Preventive Services Task Force recommends providers offer individualized healthy behavior interventions for all adults, independent of their risk of cardiovascular disease. While strong evidence exists to support disease-specific programs designed to improve multiple lifestyle behaviors, approaches to adapting these interventions for a broader population are not well established. Digital behavior change interventions (DBCIs) hold promise as a more generalizable and scalable approach to overcome the resource and time limitations that traditional behavioral intervention programs face, especially within an occupational setting.

**Objective:**

We aimed to evaluate the efficacy of a multimodal DBCI on (1) self-reported behaviors of physical activity, nutrition, sleep, and mindfulness; (2) cardiometabolic biomarkers; and (3) chronic disease–related medical expenditure.

**Methods:**

We conducted a 2-arm randomized controlled trial for 12 months among employees of an academic health care facility in the United States. The intervention arm received a scale, a smartphone app, an activity tracker, a video library for healthy behavior recommendations, and an on-demand health coach. The control arm received standard employer-provided health and wellness benefits. The primary outcomes of the study included changes in self-reported lifestyle behaviors, cardiometabolic biomarkers, and chronic disease–related medical expenditure. We collected health behavior data via baseline and quarterly web-based surveys, biometric measures via clinic visits at baseline and 12 months, and identified relevant costs through claims datasets.

**Results:**

A total of 603 participants were enrolled and randomized to the intervention (n=300, 49.8%) and control arms (n=303, 50.2%). The average age was 46.7 (SD 11.2) years, and the majority of participants were female (80.3%, n=484), White (85.4%, n=504), and non-Hispanic (90.7%, n=547), with no systematic differences in baseline characteristics observed between the study arms. We observed retention rates of 86.1% (n=519) for completing the final survey and 77.9% (n=490) for attending the exit visit.

**Conclusions:**

This study represents the largest and most comprehensive evaluation of DBCIs among participants who were not selected based on their underlying condition to assess its impact on behavior, cardiometabolic biomarkers, and medical expenditure.

**Trial Registration:**

ClinicalTrials.gov NCT04712383; https://clinicaltrials.gov/study/NCT04712383

**International Registered Report Identifier (IRRID):**

RR1-10.2196/50378

## Introduction

In July 2022, the US Preventive Services Task Force (USPSTF) recommended that providers offer personalized health behavior interventions for improving diet and physical activity to all adults in the United States, independent of an individual’s underlying risk for cardiovascular disease [1]. Tacit within this recommendation is a recognition that Americans, generally speaking, have suboptimal lifestyle behaviors [2,3] and have an increased risk of cardiovascular disease. Less than half of all Americans engage in the recommended 150 minutes per week of moderate-intensity aerobic activity or 75 minutes/week of vigorous physical activity, and only 12.3% and 10% of 294,566 surveyed adults in the United States meet the recommended daily intake of fruit or vegetables, respectively [4,5]. It is estimated that half of all American adults (117 million individuals) have at least 1 or more chronic diseases [4,6] and cardiovascular disease is the number 1 cause of death and disability [7,8]. Evidence suggests that chronic disease incidence and progression are accelerated by suboptimal health behaviors [9-12]; conversely, improving lifestyle behaviors can prevent the onset of chronic disease or modify its trajectory [13-18].

Strong evidence for the ability of an intervention to improve multiple lifestyle behaviors and prevent or delay disease onset comes from the literature surrounding the Diabetes Prevention Program, which focuses interventions among the population with prediabetes and has demonstrated as much as 30% to 60% reduction in the incidence of diabetes [19-21]. Additionally, scaling up the Diabetes Prevention Program required significant support from the Centers for Disease Control and Prevention, including increasing workforce expansion to implement the program cost-effectively, standardizing program implementation to assure quality, providing infrastructure resources to build sites that can provide the program, and developing a network to increase referrals for use of the prevention program [20,21]. However, the translation of these effects for a more generalized population is not well established [20,21]. Certainly, extending this model to all adults, independent of their risk of chronic disease, would require a huge financial and logistical undertaking, which may not be feasible.

Digital behavior change interventions (DBCIs) use mobile apps, wearables, computer programs, or websites to prevent disease through health behavior promotion and hold promise to overcome resource and time limitations that traditional behavioral interventions programs face [22-24]. Indeed, digitization of the Diabetes Prevention Program has demonstrated similar treatment effects [25-27]. Efforts to evaluate the feasibility of implementation and maintenance of in-person and digital diabetes prevention program are underway and will provide critical insight for scaling up implementation of digital approaches [28,29]. Whether the success of a digital Diabetes Prevention Program translates to the general population, that is, with and without prediabetes, remains unknown. Thus, rigorous evaluation of a holistic DBCI in the general population is needed in light of the USPSTF’s recommendation. However, investigating the effects of multimodal intervention on a diverse set of behaviors is difficult, and recruitment, retention, and DBCI adherence pose a challenge for conducting these types of studies in populations that are not recruited from clinics or on the basis of their underlying conditions.

Occupational settings provide a unique opportunity to engage a large proportion of the adult population in lifestyle behavior intervention and have been endorsed by the American Heart Association [30]. In addition to promoting healthy behavior among adults, wellness programs in the workplace can boost morale and improve productivity [31,32]. Additionally, DBCI programs are less likely to be disruptive in an occupational setting in comparison to traditional programs and, therefore, more likely to experience higher engagement and adherence. However, the availability of resources such as in-person coaching, room and space availability, and time off from work responsibilities constrain the ability to scale behavioral interventions.

To address these issues, we present a framework for a DBCI that integrates the Health Belief Model with proposed mechanisms of lifestyle interventions’ effect on chronic conditions as a precursor to subsequent research that will describe the primary outcomes of our innovative study. In this paper, we describe the design of the UMass Fitbit Care Study, which evaluates a multimodal DBCI provided by Fitbit among health care employees in Central Massachusetts. The primary objectives of the UMass Fitbit Care study were to evaluate the efficacy of intervention on the following (Textbox 1):

Our hypothesis is that self-reported lifestyle behaviors are going to be a proximal outcome affected by the intervention and likely to show the strongest treatment effect; cardiometabolic biomarkers will be more distal and show a modest treatment effect; and medical expenditure will be the most distal outcome and demonstrate a minimal treatment effect during the study period but may reveal a trend towards reduced expenditure among the intervention group.

The secondary objectives of the UMass Fitbit Care study were to characterize the following (Textbox 2):

**Primary objectives of the UMass Fitbit Care study.**

- Change in self-reported lifestyle behaviors on physical activity, nutrition, sleep, and mindfulness among participants
- Cardiometabolic biomarkers over a 12-month period
- Chronic disease–related medical expenditure

**Secondary objectives of the UMass Fitbit Care study.**

- Trajectories of intervention engagement over a 12-month period
- Differential engagement of the Fitbit intervention in relation to baseline behavior and comorbidities
- Differential efficacy of interventions based on engagement

## Methods

### Study Design

**Figure 1:**
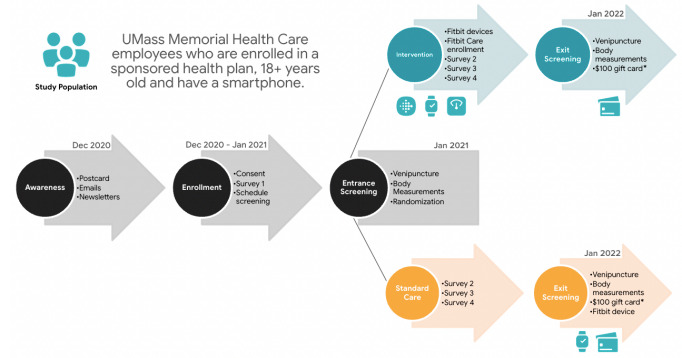
UMass Fitbit Care Study design: health behavior data were collected through baseline and quarterly web-based surveys, while biometric measurements were obtained during clinic visits at baseline and at the 12-month follow-up. The exit screening phase concluded in January 2022. *US dollars.

The UMass Fitbit Care Study is a dual-arm, unblinded randomized clinical trial with a 12-month study period. SPIRIT (Standard Protocol Items: Recommendations for Interventional Trials) reporting guidelines were used in the development of this protocol and fulfilled the SPIRIT checklist [33]. Recruitment began in December of 2020. Participants were randomized to the intervention or control arm using a permuted block randomization that stratified based on age (40 vs ≥40) and sex to achieve a balance for those 2 demographic variables between the intervention and control groups (Figure 1). Participants were randomized 1:1 to a control arm, where participants only received their standard health and wellness benefits during the study period, or an intervention arm, where participants received their standard health and wellness benefits as provided by their employer, plus a digital behavioral change intervention through Fitbit at no cost for 1 year. The Fitbit DBCI included 5 components: a wearable health and fitness device (participant choice of Fitbit Inspire 2 tracker or Versa 3 smartwatch), a wireless weight scale (Fitbit Aria Air), the Fitbit app (provided basic tracking and goal setting functionalities), Fitbit Premium (provided additional advanced metrics and rich video content through the Fitbit app), and an on-demand human health coach (provided goal setting, action planning, and accountability support through secure, asynchronous, in-app messaging).

### Study Setting and Participants

This study was conducted in Worcester, Massachusetts, with employees of UMass Memorial Health (UMMH). To be eligible, participants must have been enrolled on a UMMH-sponsored health plan, had to own a smartphone, had to be 18 years of age or older, and agreed to consent to the study (Figure 1). Participants who were pregnant at the time of enrollment, did not speak English, or were incarcerated were excluded from the study. The study had an a priori enrollment goal of up to 700 employees, which represents roughly 7.5% of the entire eligible population. The sample size for this study was derived based on the pragmatics of resource availability. All eligible employees were invited to participate in the study through electronic communication and a home mailer. To be enrolled in the study, participants had to electronically sign informed consent, fill out a baseline survey and demographics, use a self-scheduling service to pick an appointment for their initial biometric screening, and then complete said screening in person. Participants were then randomized to either the intervention arm or the control arm. Randomization was conducted using a random number generator to provide a random number of 1 or 2 for each of the study participants. A permuted block design with random block sizes was used to generate the randomization sequence. That sequence was loaded into a randomization module using a free, secure, and robust data collection platform called REDCap (Research Electronic Data Capture; developed at Vanderbilt University) so that it was readily available during the in-person screening process. Participants were asked to schedule an exit biometric screening at the end of the 12-month study period. Because this intervention being investigated is designed to help users improve their health behaviors in alignment with the advice of their health care providers and not to make medical decisions, the risk of harm to health is low. Participants were free to withdraw from the study at any time.

### Ethical Considerations

A trial steering committee consisting of 2 principal investigators, JM and SB, met biweekly to provide supervision of the study, ensure progress milestones were met, and communicate important protocol updates to relevant parties. The study was reviewed and approved by the University of Massachusetts Chan Medical School (UMass Chan) Institutional Review Board (approval #H00021669) and registered on ClinicalTrials.gov (NCT04712383) on January 13, 2021. All participants had to electronically sign an informed consent to participate in the study. Entrance and exit biometric exam data were collected in person; held by Quest Diagnostics; and electronically transferred to UMass Chan, where it was stored on an encrypted server approved for data containing personal health information and personal identifying information. The medical claims data from Conifer were also transferred to the same server for analysis. Participants who completed both the entrance and exit biometric screenings were provided a US $100 incentive via a Visa e-gift card. Additionally, participants assigned to the intervention arm were able to keep the Fitbit wearable device and weight scale received during the study, and those assigned to the control arm received a Fitbit wearable device at their exit screening appointment.

### Intervention

Participants in the intervention group were provided with a Fitbit tracker and scale and received assistance in creating a Fitbit account in the Fitbit app, syncing their devices, and enrolling into the study experience in the Fitbit app. Enrolling into the study experience gave each participant access to Fitbit Premium and Health Coaching.

### Fitbit DBCI Components

#### Wearable Health and Fitness Device

Intervention participants had a choice of a Fitbit Inspire 2 tracker or a Fitbit Versa 3 smartwatch. Both devices provided comparable health and fitness tracking features and capabilities, including steps, distance, energy burn, exercise, heart rate, active zone minutes (AZM; measure of moderate-to-vigorous physical activity based on the Physical Activity Guidelines for Americans), and sleep. The Fitbit Inspire 2 had a smaller, bracelet-like form factor and longer battery life (~10 days), while the Fitbit Versa 3 had a watch form factor, additional smartwatch features (built-in GPS, apps, etc.), and a shorter battery life (~6 days).

#### Wireless Scale

The Fitbit Aria Air scale provided intervention participants with a simple wireless (Bluetooth) scale that integrated with the Fitbit app for ease of weight trend tracking.

#### Fitbit App

The Fitbit app provided intervention participants with features to enhance tracking and goal setting for each of the health and fitness measures tracked by their wearable device. For example, the AZM feature in the app allowed users to see graphs of their trends in moderate-to-vigorous physical activity over time (daily, weekly, monthly, etc.) and to set personal goals for the number of AZMs to achieve in a day and a week. As another example, the sleep tracking feature allowed them to see their sleep duration, bedtime and wake-up time consistency, and time spent in different sleep stages and to see trends in these measures over time, as well as set goals for sleep duration, bedtime, and wake-up time.

#### Fitbit Premium

**Figure 2:**
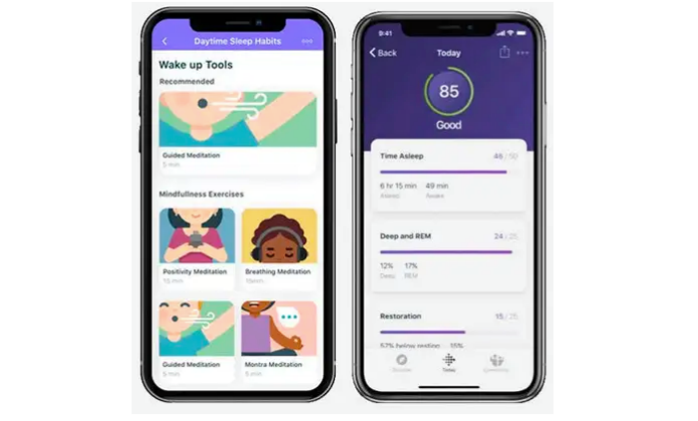
Sample screenshots of Fitbit Premium features: rich video content including meditation videos (left) and advanced analytics including sleep score (right).

Participants assigned to the intervention arm also received Fitbit Premium. Fitbit Premium is a subscription membership service that provides advanced scores and rich content within the Fitbit app to enhance the participant experience. The advanced scores, included in both the sleep score and stress management score, provide additional feedback and insights to users on these dimensions of their wellness. The rich content includes hundreds of workout videos and mindfulness audio sessions, as well as healthy recipes, all designed to help users improve their health behaviors (Figure 2).

#### On-Demand Human Health Coach

**Figure 3:**
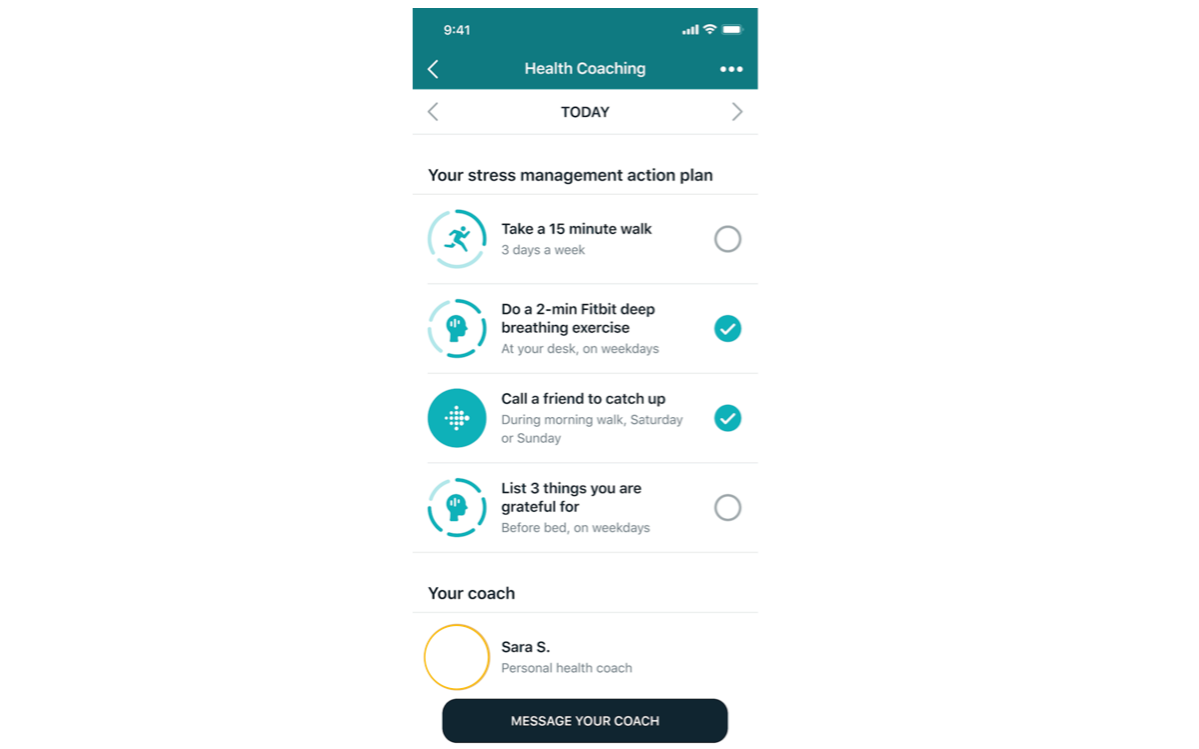
Sample screenshot of on-demand health coaching features. The participant and coach collaboratively create an action plan for stress management. The user can click on the coach’s profile to learn more about their background or click on the messaging button to chat securely and asynchronously.

Each intervention participant was paired with a personal, human health coach that provided goal setting, action planning, and accountability support for health behavior improvement across activity, nutrition, sleep, and mindfulness, with emphasis on the domains desired by the participant at any moment in time. The health coaches sent a secure, asynchronous, in-app SMS text message within a day of enrollment to each participant to start the process of creating a goal and an action plan for improving the behaviors when the participant’s motivation and confidence were greatest. For example, there might be an exchange of a few messages to determine that a particular user wanted to focus on improving sleep. The coach might help that user set a goal about a regular sleep schedule and an action plan for doing a mindfulness session before bed and not bringing electronic devices to bed (Figure 3).

The health coach then sent a message every day or every other day for the first few weeks to encourage the participant to stick with the plan and to provide support for follow-through or adjustment of the plan. The health coaches monitored core Fitbit data from the users (steps, AZMs, sleep, etc) via a dashboard to help tailor their support. The frequency of messages decreased as the participant gained self-efficacy; however, the option to increase messaging remained if the participant wanted extra support. Each participant was free to use any of the software features of the Fitbit app and Fitbit Premium as desired throughout the course of the intervention, although the health coach made recommendations on features such as workout videos, mindfulness sessions, etc that might be most helpful based on the participant’s goals, action plan, and device data. If participants were unresponsive for several weeks or requested to discontinue coaching, the health coaches archived their profiles in their dashboard and ceased sending them messages unless the participant proactively re-engaged by sending the coach a message.

### Data Collection and Management

There were four forms of data collected: (1) survey data through baseline surveys and scheduled quarterly surveys for all participants; (2) Fitbit-tracked data for those participants assigned to the intervention arm; (3) biometric data for all participants, which include anthropometric and vital sign measurements and laboratory investigations performed by Quest Diagnostics; and (4) total medical expenditure claims data abstracted by Conifer Health Solutions for all participants. Table 1 describes all the data collected as part of this study. An initial invitation to participate in the study was sent via email to a list of predetermined eligible employees. Interested participants signed up for the study by entering their email address, signing the consent form, and filling out their baseline survey and demographics and presented at an in-person biometric screening appointment performed by Quest Diagnostics staff. At this first visit for the biometric screening, participants in both the control group and intervention group were randomized and scheduled for the 4 follow-up quarterly surveys to study trends in perceptions, motivation, and confidence around improvements in health behaviors. An email was sent to participants each quarter with a link to the quarterly survey in UMass REDCap. Up to 2 reminder emails were sent to participants who did complete the survey. An additional email to all participants was sent in September 2021 to re-engage participants at the three-quarters point of the study. All survey data collection was performed using the REDCap data collection platform. SAS (SAS Institute) was used for all analyses. The final trial dataset, which consists of survey questionnaire data, Fitbit device data, Quest biometric data, and Conifer claims data, is only available to partners within the UMass Chan Department of Population and Quantitative Health Sciences who were responsible for the analysis.

**Table 1:** Data collection schedule.

Events and procedures	Study period (January-December 2021)									Poststudy period
	Enrollment	Allocation	Postallocation				Close-out			
	Visit 0	Visit 1	3 months	6 months	9 months	12 months or visit 2				
Enrollment										
Informed consent	✓									
Demographics	✓									
Eligibility confirmation	✓									
Allocation		✓								
Assessment										
Biometric screening (height, weight, waist circumference, blood pressure, etc)—Quest Diagnostics		✓				✓				
Baseline survey (nutrition, exercise, sleep, and mindfulness habits)—REDCap^a^	✓									
Quarterly survey (nutrition, exercise, sleep, and mindfulness habits)—REDCap			✓	✓	✓	✓				
Two years of total medical expenditure claims data—Conifer Health Solutions								✓		
Intervention										
Onboarding survey for coaching component		✓								
Engagement data for each component of intervention		Continuous collection of device wear duration Event-based collection of app-open events, feature usage (including Fitbit Premium features), secure messages with coach, etc	Continuous collection of device wear duration Event-based collection of app-open events, feature usage (including Fitbit Premium features), secure messages with coach, etc	Continuous collection of device wear duration Event-based collection of app-open events, feature usage (including Fitbit Premium features), secure messages with coach, etc	Continuous collection of device wear duration Event-based collection of app-open events, feature usage (including Fitbit Premium features), secure messages with coach, etc	Continuous collection of device wear duration Event-based collection of app-open events, feature usage (including Fitbit Premium features), secure messages with coach, etc				
Wearable device and weight scale data		Continuous collection of wearable device data (steps, resting heart rate, sleep metrics, etc) Event-based collection of weight data	Continuous collection of wearable device data (steps, resting heart rate, sleep metrics, etc) Event-based collection of weight data	Continuous collection of wearable device data (steps, resting heart rate, sleep metrics, etc) Event-based collection of weight data	Continuous collection of wearable device data (steps, resting heart rate, sleep metrics, etc) Event-based collection of weight data	Continuous collection of wearable device data (steps, resting heart rate, sleep metrics, etc) Event-based collection of weight data				

^a^REDCap: Research Electronic Data Capture.

### Statistical Analysis

In this paper, we present descriptive statistics for baseline characteristics and overall engagement among the participants assigned to the intervention group using proportions and the mean for the distribution of categorical and continuous variables, respectively. The study will evaluate its primary objectives using intention-to-treat analysis. A 2-tailed Student *t* test for independent samples will be used to compare the mean of continuous variables, and a chi-square or Fisher exact test will be used to compare categorical variables between the intervention and control groups. Secondary objectives will be evaluated using group-based trajectory modeling to identify distinct trajectories of engagement and adherence, the association of distinct trajectories with baseline behavior and comorbidities, and performing an intervention-only analysis where the treatment effect of the intervention is evaluated across different trajectories of engagement. Additional exploratory analyses will consider accounting for possible contamination based on the self-reported use of wearable health and fitness devices among the control group participants. There are no plans to use biological specimens in ancillary studies. Data collection questionnaires can be provided upon request.

## Results

### Baseline Characteristics

A total of 13,391 employees were eligible to enroll in the UMass Fitbit Care Study and received an email or home mailer about participating in the study. Of those invited, 758 completed consent to participate in less than 24 hours and scheduled an appointment for initial biometric screening. The follow-up rate was 75.7%, with 603 individuals attending the biometric screening and getting randomized to the intervention (n=300, 49.8%) or control group (n=303, 50.2%). Baseline characteristics of these 2 groups are provided in Table 2, and no systematic differences were observed between the 2 groups. On average, participants were 46.7 (SD 11.2) years of age. The majority of the participants were female (80.3%, n=484), White (85.4%, n=504), and non-Hispanic (90.7%, n=547). More than half (74.8%, n=440) of the participants had a baseline BMI that classified them as overweight or obese.

**Table 2:** Baseline participant characteristics.

Characteristics		Intervention (n=300)	Control (n=303)	*P* value	
**Age (years), mean (SD)**		46.3 (11.0)	47.2 (11.4)	.34	
**Sex, n (%)**					.50
	Female	241 (80.3)	243 (80.2)		
**Race (intervention: n=295; control: n=295), n (%)**					.80
	White	254 (86.1)	250 (84.8)		
	Black or African American	11 (3.7)	9 (3.1)		
	Asian	20 (6.8)	22 (7.5)		
	Other	10 (3.4)	14 (4.8)		
**Ethnicity, n (%)**					.34
	Hispanic	16 (5.3)	25 (8.3)		
	Non-Hispanic	277 (92.3)	270 (89.1)		
	No answer	7 (2.3)	8 (2.6)		
**BMI classification (intervention: n=297; control: n=291), n (%)**					.56
	Underweight	3 (1.0)	2 (0.7)		
	Normal	65 (21.9)	78 (26.8)		
	Overweight	100 (33.7)	91 (31.3)		
	Obese	129 (43.4)	120 (41.2)		
**Biometric measurements, mean (SD)**					
	Weight (lb; intervention: n=297; control: n=292)	185.5 (46.9)	178.4 (43.7)	.06	
	Height (in; intervention: n=297; control: n=291)	65.5 (3.5)	65.2 (3.3)	.26	
	BMI (kg/m^2^; intervention: n=297; control: n=291)	30.4 (7.1)	29.5 (6.7)	.12	
	Waist circumference (in; intervention: n=294; control: n=289)	37.6 (6.5)	36.4 (6.0)	.02	
	Systolic BP^a^ (mm Hg; intervention: n=297; control: n=291)	121.0 (9.6)	119.2 (10.2)	.03	
	Diastolic BP (mm Hg; intervention: n=297; control: n=291)	75.2 (8.0)	74.3 (7.9)	.17	
	HbA_1c_^b^ (%; intervention: n=297; control: n=292)	5.2 (0.6)	5.2 (0.5)	.47	
	Total cholesterol (mmol/L; intervention: n=296; control: n=291)	190.6 (38.7)	196.8 (38.0)	.05	
	HDL-C^c^ (mmol/L; intervention: n=296; control: n=292)	58.0 (14.5)	59.3 (14.7)	.29	
	LDL-C^d^ (mmol/L; intervention: n=296; control: n=292)	118.7 (34.5)	123.5 (34.6)	.10	
	TC/HDL ratio^e^ (intervention: n=296; control: n=292)	3.5 (1.0)	3.5 (1.2)	.57	

^a^BP: blood pressure.

^b^HBA_1c_: hemoglobin A_1c_.

^c^HDL-C: high-density lipoprotein cholesterol.

^d^LDL-C: low-density lipoprotein cholesterol.

^e^TC/HDL ratio: total cholesterol/high-density lipoprotein cholesterol ratio.

### Intervention Engagement and Participant Retention

**Figure 4:**
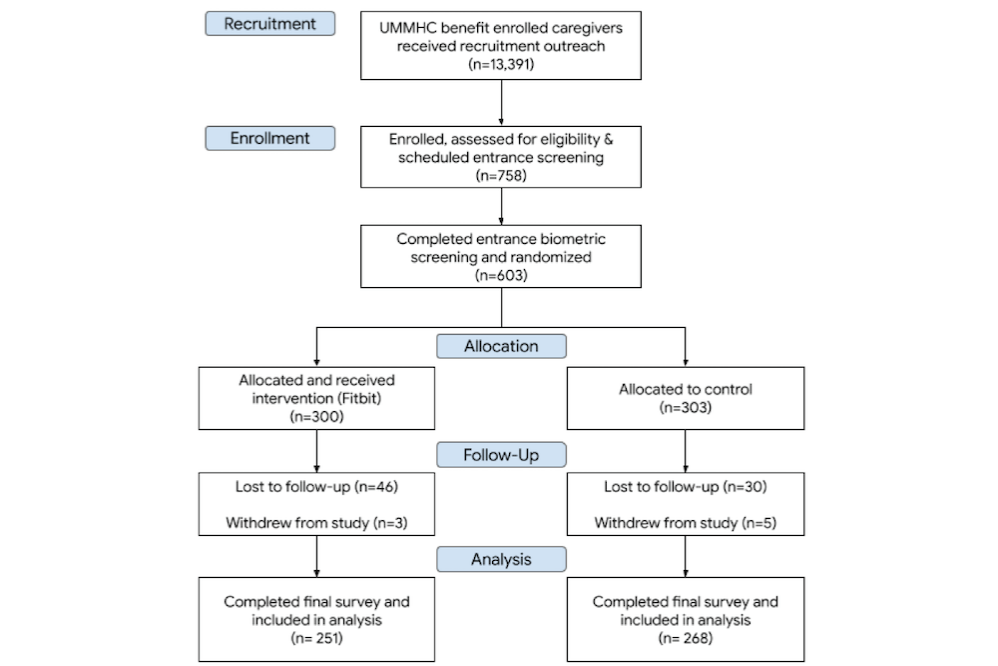
UMass Fitbit Care Study participant flow: flow of participants through the UMass Fitbit Care study, showcased by the CONSORT (Consolidated Standards of Reporting Trials) diagram. UMMHC: UMass Memorial Health Center.

To approximate real-world scenarios, this study did not have a specific plan to promote participant engagement with the intervention or retention of participants in the study beyond ensuring that their Fitbit device was synced with the mobile app and initial communication with their personal health coach occurred. The median wear time for Fitbit was observed to be 325 (IQR 154-372) days, and 264 (88%) participants used health coaching at least once. Of the 603 participants who completed entrance screening and thus were included in the study, 8 (1.3%) withdrew, 519 (86.1%; intervention: n=251; control: n=268) completed exit surveys, and 469 (77.8%; intervention: n=227; control: n=243) completed exit biometric screening (Figure 4).

## Discussion

### Overview

We describe the design, rationale, and baseline characteristics of a randomized control trial of a multimodal DBCI from Fitbit on improving lifestyle behaviors and cardiometabolic health in a general, rather than disease-specific, population. The high retention rate and balanced distribution of baseline characteristics, paired with a comprehensive assessment of the intervention’s impact on behavioral, cardiometabolic biomarkers, and medical expenditure, will allow for detailed evaluation of the intervention for primary and secondary endpoints.

### Comparison With Prior Work

Research on the effectiveness of DBCIs for chronic disease prevention has yielded mixed results with regard to behavioral change and cardiometabolic outcomes. One randomized controlled trial led by Toro-Ramos et al [34] assessed the effectiveness of a lifestyle app in individuals with prediabetes, in which significant weight loss was observed in the intervention group, but there was no significant difference in hemoglobin A_1c_ levels between the intervention and control groups at 1 year (mean difference 0.006%; n=103; *P*=.93) [35]. Likewise, another study investigating a mobile diabetes prevention program found a 1.9% (n=163) decrease in hemoglobin A_1c_ among intervention participants compared to the control group, but there were no improvements in patient-reported behaviors such as mood after 12 months [36]. Additionally, such interventions have often focused on evaluating behaviors within disease-specific populations [34,37-40].

### Future Aims

Therefore, we aim to adopt a more holistic approach to behavior change intervention research by evaluating the effectiveness of a DBCI intervention in a broader, real-world population and assessing changes in behavior across 4 key areas: physical activity, nutrition, sleep, and mindfulness. Additionally, we will conduct comprehensive biometric evaluations using both digital and laboratory biomarkers, which will contribute valuable insights to the existing literature.

**Figure 5:**
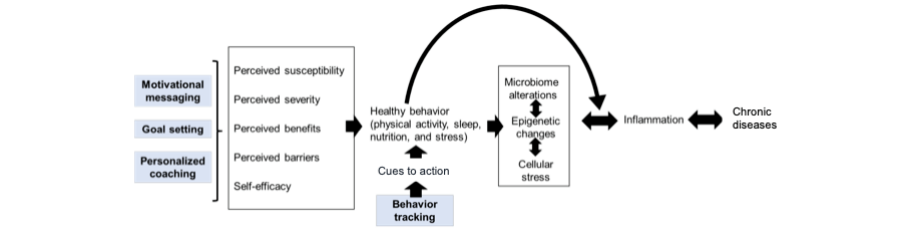
Conceptual framework of Fitbit’s multimodal digital behavior change intervention (DBCI) for improving healthy behaviors and cardiometabolic outcomes. Components of Fitbit’s DBCI are depicted in blue boxes. The framework represents a combination of the Health Belief Model and the framework proposed by Bodai et al.

There are several potential mechanisms through which Fitbit DBCI can affect healthy behavior changes, which lie upstream of factors that impact the onset and progression of chronic diseases. The Lifestyle Medicine Research Summit proposed that promotion of healthy lifestyle behavior can interrupt the pathogenesis of chronic diseases and possibly reverse it [41-44]. The Health Belief Model is a well-established theory to explain an individual’s decision-making for a behavioral change [45]. We propose that an integration of these 2 frameworks, as described in Figure 5 [43], can provide theoretical underpinning for the impact of the Fitbit DBCI on chronic diseases. Fitbit DBCI’s ability to pair tracking of behavior with nudges and coaching that can be delivered through telecommunications and motivational messaging can act on lifestyle behaviors that lie upstream of factors that lead to chronic diseases [37,46-50]. Support for individual goal setting and self-monitoring through alerts based on certain patterns of inactivity or pushing motivational messages based on the accomplishment of healthy behaviors can motivate individuals, promote self-efficacy, and provide cues for action [51-53]. It offers a holistic approach to improving lifestyle behaviors and is responsive to the USPSTF’s recommendation for tailoring behavioral intervention to individuals’ needs.

In our study, we observed a high adherence to wearable devices, with a median wear time of 325 (IQR 154-372) days. We had an 86.1% (n=519) retention rate for exit surveys that evaluated self-reported behavioral outcomes and a 77.9% (n=490) retention rate for exit visits that measured cardiometabolic biomarkers. Our results are consistent with findings from prior research on DBCIs for diabetes and hypertension prevention [26,39].

Further secondary analysis of the data from this study can inform the feasibility and acceptance of providing multimodal DBCIs for employees in a workplace setting. Additionally, through the collection of data over the course of the year from the Fitbit intervention, we will be able to perform secondary data analysis that characterizes the intensity of the coaching intervention, which is likely to be a key determinant of its efficacy. A 3-year study showed that individualized biweekly counseling decreased sedentary time and promoted healthy behavior [41,42]. However, it is unlikely that all users of the program require an intervention of this intensity. We will be able to perform analysis to understand factors associated with goal setting and the self-efficacy of participants for affecting behavioral change as measured through standardized questionnaires. Thus, our findings will inform optimal intervention practices for implementing DBCI programs at scale, where participant-reported outcomes can be used to facilitate tailored behavioral counseling for participants and align DBCI programs with USPSTF recommendations for individualizing interventions.

### Strengths and Limitations

Several aspects of the study represent methodological strengths, which include the use of a randomized design, rigorous and standardized outcome evaluation, and robust adherence to the protocol. However, the study’s limitations should also be noted when interpreting the results from this study. Our study lacks an active control and therefore we cannot estimate treatment effects of activity tracking and DBCI separately. However, the intended use of Fitbit health coaching is as a combination, and therefore, this study estimates the combined treatment effect of this multimodal intervention. Our study sample underrepresents Black or African American, Hispanic, and Asian staff, suggesting a need for future studies to implement robust recruitment strategies to target underrepresented groups. Moreover, our study sample was selected from employees at an urban academic medical center, which may suggest that these participants are already engaging in healthy behaviors given their health backgrounds. Our study sample included individuals who volunteered to participate in digital health research. Our baseline characteristics suggest that there is skewness in demographic representation such that individuals more likely to participate in these studies are enrolled in this study, which may account for our high adherence and retention rates. While that limits the generalizability of the study findings, the randomized assignment of interventions balances any possible bias that may emerge from early responders to the invitation. Lifestyle behavior was measured using standardized surveys with high face validity, but these were not validated instruments, such as the Pittsburgh Sleep Quality Index, 24-hour food recall, perceived stress scale, etc, which limits the external generalizability of the findings; however, differences between groups in these measures are unlikely to be affected by them.

### Conclusions

To our knowledge, this is one of the largest evaluations of a multimodal DCBI’s impact on physical activity, nutrition, sleep, and mindfulness among participants who were recruited independent of their underlying disease status. It is also among the only studies that comprehensively evaluates the impact of the DBCI on the sequelae of healthy behavior by measuring cardiometabolic biomarkers and abstracting medical expenditure data. Finally, data from this study can help understand adherence to DBCIs over the period of 1 year and elucidate the association between intensity of coaching, self-reported motivation for changing behavior, and objective measurement of health behavior trajectories from wearable health and fitness devices and wirelessly connected scales. Taken together, the findings from this study have the promise to provide useful insights for individualizing the Fitbit intervention among otherwise healthy people, as recommended by the 2022 USPSTF.

## Appendix

Multimedia Appendix 1 SPIRIT (Standard Protocol Items: Recommendations for Interventional Trials) checklist.

Multimedia Appendix 2 CONSORT-EHEALTH (V 1.6.1) checklist.

## Abbreviations

AZM: active zone minutes
CONSORT: Consolidated Standards of Reporting Trials
DBCI: digital behavior change intervention
REDCap: Research Electronic Data Capture
SPIRIT: Standard Protocol Items: Recommendations for Interventional Trials
UMass Chan: University of Massachusetts Chan Medical School
UMMH: UMass Memorial Health
USPSTF: US Preventive Services Task Force

## Data Availability

The datasets analyzed during this study are available from the corresponding author on reasonable request. Due to the collection of participant health information, we do not have plans for granting public access to participant-level datasets.
